# Skin senescence—from basic research to clinical practice

**DOI:** 10.3389/fmed.2024.1484345

**Published:** 2024-10-18

**Authors:** Natalia Dorf, Mateusz Maciejczyk

**Affiliations:** ^1^Independent Laboratory of Cosmetology, Medical University of Białystok, Bialystok, Poland; ^2^Department of Hygiene, Epidemiology and Ergonomics, Medical University of Białystok, Bialystok, Poland

**Keywords:** skin, aging, skin aging, senescence, biomarkers

## Abstract

The most recognizable implications of tissue aging manifest themselves on the skin. Skin laxity, roughness, pigmentation disorders, age spots, wrinkles, telangiectasia or hair graying are symptoms of physiological aging. Development of the senescent phenotype depends on the interaction between aging cells and remodeling of the skin’s extracellular matrix (ECM) that contains collagen and elastic fiber. Aging changes occur due to the combination of both endogenous (gene mutation, cellular metabolism or hormonal agents) and exogenous factors (ultraviolet light, environmental pollutants, and unsuitable diet). However, overproduction of mitochondrial reactive oxygen species (ROS) is a key factor driving cellular senescence. Aging theories have disclosed a range of diverse molecular mechanisms that are associated with cellular senescence of the body. Theories best supported by evidence include protein glycation, oxidative stress, telomere shortening, cell cycle arrest, and a limited number of cell divisions. Accumulation of the ECM damage is suggested to be a key factor in skin aging. Every cell indicates a functional and morphological change that may be used as a biomarker of senescence. Senescence-associated *β*-galactosidase (SA-β-gal), cell cycle inhibitors (p16INK4a, p21CIP1, p27, p53), DNA segments with chromatin alterations reinforcing senescence (DNA-SCARS), senescence-associated heterochromatin foci (SAHF), shortening of telomeres or downregulation of lamina B1 constitute just an example of aging biomarkers known so far. Aging may also be assessed non-invasively through measuring the skin fluorescence of advanced glycation end-products (AGEs). This review summarizes the recent knowledge on the pathogenesis and clinical conditions of skin aging as well as biomarkers of skin senescence.

## 1 Introduction

All organs in the human body are subject to a complex aging process that may be described as a progressive loss of cell functionality and regenerative potential. The most recognizable and visible implications of body aging manifest themselves on the skin. Skin is the largest human organ that has an approximate surface area of 2 m^2^ in an adult (1). Depending on the anatomical location its thickness varies from 0.8 to 5 mm (2). Aging involves the cellular and histological changes of epidermis, dermal-epidermal junction and dermis. That multilayered construction is tightly related to skin functions. Skin is an area of constant interaction between the internal and external environment. Its primary function is to protect the body from harmful external agents, including physical [heat, cold, ultraviolet light (UV)], biological (bacteria, vi-ruses, pathogenic fungi) and chemical (harmful acids and detergents) factors. Skin also regulates body temperature, prevents water loss or receives stimuli from the external environment (1–3).

The process of skin aging consists of various interdependent characteristics at the molecular, cellular, and organ levels. Cellular senescence is the primary aging process at a cellular level, associated with a loss of proliferative capacity. Senescence of dermal fibroblasts and epidermal keratinocytes is the main cause of skin aging that results in a decline in the integrity and function of the skin. A range of diverse molecular mechanisms is associated with skin aging. Emerging hypotheses postulate that fibroblast senescence is associated with an irreversible proliferation arrest and an enhanced release of the senescence-associated secretory phenotype (SASP). Aging theories best supported by evidence include protein glycation, oxidative stress, and telomeres shortening (4, 5). It is suggested that overproduction of mitochondrial reactive oxygen species (ROS) is a key factor driving the cellular senescence (5).

Long-term exposure to external factors affects biochemical, structural and physical properties of the skin, causing premature aging. Extrinsic aging, known as photoaging, results from the skin exposure to various environmental factors, including UV radiation, pollution, and smoking. In the meantime, the human body undergoes a genetically determined, naturally occurring process known as chronological aging. Skin aging manifests itself as dryness, laxity, and elastosis of the skin; an increased number of wrinkles, telangiectasia, and aberrant pigmentation of the skin (3, 6). The accumulated damage to the extracellular matrix (ECM) components constitutes a key factor in the typical signs of aging: deep wrinkles on the forehead, around the lips and eyes, a prominent nasolabial groove, and sagging excess skin (7).

The primary method of defining human age is to count the number of years one has lived, known as chronological age. Actually, a more specific concept of passing the time is biological aging, also known as physiological or functional aging. The difference between both types of aging is that biological aging accounts for not only the time elapsed but especially refers to how old cells and tissues in the entire body are (8). It takes into account biological and physiological factors, such as a lifestyle or nutrition, that influence the human body to a great extent. If somebody is healthy and fit for his or her age, it means that his/her biological age may be lower than the chronological age. Aging causes every cell to indicate a functional and morphological change that may be used as a biomarker of senescence. Such biomarkers allow to understand how advanced aging changes are in the entire body. All cellular hallmarks of aging are strongly related with one another: DNA instability, telomere attrition, accumulation of lipofuscin and senescence-associated *β*-galactosidase (SA-β-gal), mitochondrial dysfunction, stem cell exhaustion, inhibition of proliferation, formation of DNA damage response (DDR) and DNA segments with chromatin alterations reinforcing senescence (DNA-SCARS), senescence-associated heterochromatin foci (SAHF) constitute just examples of aging biomarkers in cells, including skin cells (8–11). This review summarizes the recent knowledge on the pathogenesis and clinical conditions of skin aging as well as biomarkers of skin senescence.

## 2 Skin structure

The epidermis, as the outermost layer of the skin, is a constantly renewing multilayered epithelium undergoing keratinization (12). *Stratum basale* is the deepest layer of the epidermis and consists of a single row of mitotically active cells with centrally located, elongated cell nuclei. Keratinocytes divide and differentiate to form new cells that move up to the skin surface to exfoliate. It takes 28–30 days to ensure a complete epidermis renewal. Besides keratinocytes, the proliferative layer also contains melanocytes, Langerhans cells and Merkel cells (2, 13). The *Stratum spinosum*, that is the layer above the stratum basale, consists of 5–15 layers of more flattened keratinocytes, each with large, rounded cell nuclei (2). The *Stratum granulosum* consists of two or three layers of spindle-shaped cells with flattened nuclei and keratohyalin granules in the cytoplasm. After the cell nucleus dies, keratohyalin granules begin to form keratins that help fill the cell’s structure. Profilaggrin, a precursor form of filaggrin, is a highly phosphorylated protein that occurs in keratohyalin granules in the granular layer. During the terminal differentiation of epidermal cells into cornified cells, profilaggrin is broken down into multiple filaggrin monomers. In proteolysis and dephosphorylation reactions, monomers are degraded into free amino acids that are then released into the cytoplasm. Amino acids are components of the natural moisturizing factor (NMF) ensuring proper skin hydration. In the meantime, lipid granules known as lamellar bodies (LGs) produce intercellular lipids to seal the epidermis. Both structures contribute to the overall hardness of the epidermis and help prevent water loss from the body (14–16). The *Stratum lucidum* is found only in the areas that are most thickened and exposed to pressure or friction, such as surface of the hands and feet (2). The *Stratum corneum* is composed of corneal plates—flat, closely arranged cells, out of organelles, filled with keratin filaments. In the most superficial layer—the exfoliating layer, cells separate from one another due to the degradation of desmosomes (2, 14).

The dermal-epidermal junction (DEJ) has a wavelike, undulation structure. It is formed by epidermal protrusions projecting more deeply into the dermis (rete ridges) and dermal elevations up into the epidermis (dermal papillae). The DEJ consists of collagen type IV, laminins, nidogens, and the heparan sulfate proteoglycan known as perlecan. That wavelike structure prevents delamination and ensures the diffusion of nutrients from the connective tissue to the epithelium (17, 18).

The dermis is made up of connective tissue elements. The three components of the connective tissue are cells, ground substance, and fiber. The main cells of the dermal connective tissue are fibroblasts. They produce components of the skin’s extracellular matrix (ECM), including the ground substance and fiber. They maintain the correct hydration and structure of the connective tissue. The ground substance is made of glycosaminoglycans (GAGs), such as chondroitin sulfate, dermatan sulfate, keratan sulfate, heparan sulfate, heparin, and hyaluronic acid (HA). HA with the greatest hygroscopic properties is the most numerous GAG in the skin dermis (19, 20). Collagen fiber, as complex *α*-helix molecules, provide the proper strength and stiffness of the tissue. In 80% of the adult dermis, type I collagen is predominant, while type III collagen, that is thinner, accounts for about 15% (21, 22). Elastic fiber is the thinnest fiber and is able to stretch many times its length, and return to its original configuration when the deformation force is removed, providing the tissue with proper elasticity (23, 24). Elastic fiber is composed of extensively cross-linked elastin (>90%). It is a long-lived protein that degrades slowly with age. Elastin is surrounded by elastic microfibrills that contain numerous proteins, especially type I fibrillin (25). In young skin, the elastic fiber is arranged in parallel and perpendicular arrays, creating a characteristic, highly ordered architecture (26). All those ECM elements are located in two structurally different dermis layers of the dermis called the papillary and reticular layers. The papillary layer contains dermal papillae formed by more loosely packed connective tissues, consisting of a small amount of collagen and a few fiber, but a large amount of GAGs (10). In papillary dermis the fibrillin-rich microfibrills of elastic fiber, known as oxytalan fiber, run perpendicularly to the skin surface (27). The papillary dermis is separated from the reticular one by the superficial capillary plexus (SCP) where the arterial network intertwines with the venous network (2, 22). The deeper reticular layer contains dense connective tissues primarily composed of type I collagen that forms thick fiber (2). Elaunin fiber, a large-diameter elastic fiber in the reticular dermis, is primarily comprised of elastin, and is horizontally arranged within the skin structure (27, 28). Together with elastin strands, they create an organized, compressed network. A deep capillary plexus of arteries and blood veins runs at the border of the dermis and subcutaneous tissue (22).

The hypodermis, also known as subcutaneous tissue, is the innermost layer of the skin. The hypodermis is composed of a large number of adipocytes—fat cells surrounded by connective tissues. It is the major store of high-energy compounds accumulated in the form of triglycerides. It also provides mechanical protection and thermal insulation (2). The main form of adipose tissue in the skin is known as dermal white adipose tissue (dWAT). White adipocytes have a simple structure. They consist of a single large fat droplet surrounded by a few cellular organelles. That morphological structure ensures that WAT is used for energy storage purpose and forms the majority of the body fat mass. According to its distribution, WAT can be classified into two main depots: visceral WAT (VAT) and subcutaneous WAT (SAT) that is located under the skin. Only 1 to 2% of the total body fat is represented by brown adipose tissue (BAT) (29). Brown adipocytes contain numerous of small fat droplets and a high number of cellular organelles, including mitochondria. Its primary function is thermoregulation (30).

## 3 External and internal skin aging drivers

Aging is a natural stage of life. That process consists of a set of progressive changes over time, mainly in the metabolism and physicochemical properties of the cells, which leads to an impairment of the body’s self-regulation and regeneration capacity. Aging occurs mainly in the structures responsible for facial anatomy formation: skin, soft tissues, and bones (7). With the progression of aging, eyebrows start dropping, eyelids become more flaccid, and the overall eye size seems to decrease. Deep wrinkles appear on the forehead, around lips and eyes, the nasolabial groove is more prominent, and excess skin begins to sag and lose the contours of the jaw. When aging proceeds, wrinkles become evident even at rest, and excess chin fat disturbs the face shape much more. Fat atrophy, ECM remodeling and epidermal thinning proceed (7, 31). Depending on the cause, there are two main types of skin aging: intrinsic and extrinsic. Both types of skin aging share common biological, chemical, and molecular mechanisms. Those mechanisms interact with one another and together affect the overall appearance of the aging skin, which is also called true aging. Accumulation of the macromolecular damage inside and outside of cells persists over years, altering the cellular function (3). Table 1 shows the factors influencing both processes.

**Table 1:** Causes of skin aging.

Intrinsic aging (natural or chronological aging)	Extrinsic aging (photo-aging)
Passage of time	UVA/UVB and ionizing radiation
Genetic conditions	Unhygienic lifestyle
Hormonal imbalances	Unsuitable diet
Harmful effects of ROS activity	Dehydration
Immune system disorders	Environmental pollution
	Cigarette smoke
	Sleep deficiency and stressful lifestyle
	Improper skin care

ROS, reactive oxygen species; UVA, long-wave ultraviolet radiation; UVB, medium-wave ultraviolet radiation.

### 3.1 Intrinsic factors driving skin aging

**Figure 1:**
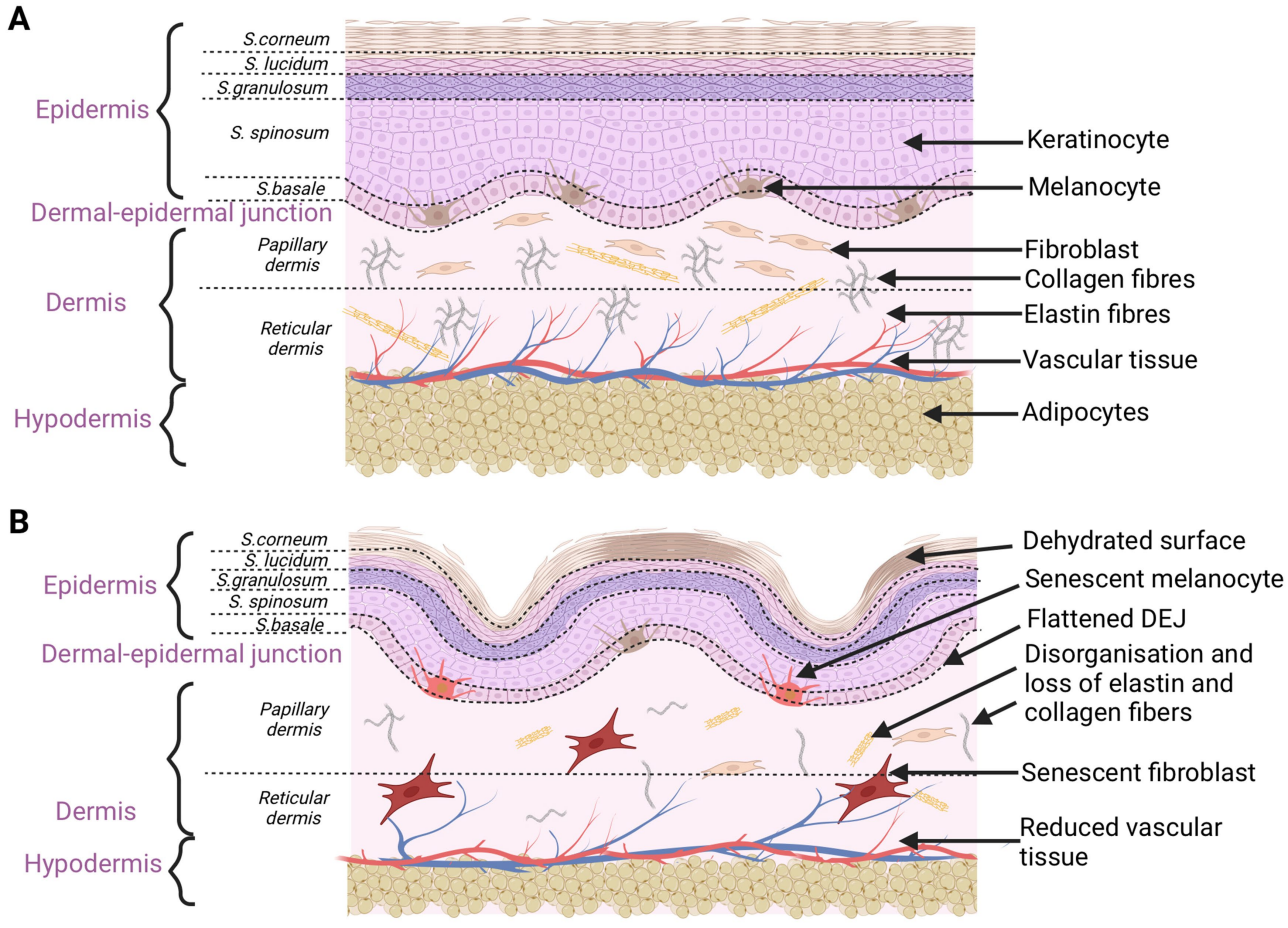
Schematic characteristics of young **(A)** and old **(B)** skin. Aging changes concern the epidermis, dermal-epidermal junction, dermis, and hypodermis. Disorganization and damage to every skin level affect the overall skin appearance (created with BioRender.com).

Intrinsic aging process is a normal, chronological aging process not only of the skin but all the body organs. A number of disturbances occur in the skin at the time, triggering a senescent state. DNA damage, telomere shortening, reactive oxygen species (ROS) overproduction, or mitochondrial dysfunction cause senescent cells to accumulate within tissues (32). There is some evidence that only 3% of intrinsic factors trigger visual changes when it comes to skin aging (31). External factors are much more responsible for that process. In the chronologically aging skin, there are some functional changes in both the epidermis, the dermal-epidermal junction and the dermis. First of all, there is a decrease in the activity of the basal layer cells at the epidermal level. Keratinocyte stem cells lose their proliferative capacity, leading to reductions in the thickness of the epidermis. Thus, epidermal abilities to regenerate is slower than in young skin. Skin thinning is the most remarkable sign of intrinsic skin aging (8). It also includes markedly declined levels of structural proteins for the epidermal permeability barrier, including filaggrin and loricrin (33). Deficiencies in those proteins change the natural barrier function of the epidermis and stratum corneum. Trans-urocanic acid and pyrrolidone carboxylic acid are the examples of filaggrin metabolites that are natural skin moisturizers (33). The reduced content of amino acids and lipids of the stratum corneum also leads to the epidermis dehydration. Skin hydration decreases with aging in the case of poor secretory functions of the sweat and sebaceous glands, making deficiency in the content of sebum and glycerol. In the epidermis, the number and activity of melanocytes changes. Their irregular architectural pattern leads to hypo- and hyperpigmentation visible on the skin surface (11, 33). Flattening of the dermal-epidermal junction is also seen in the chronologically aged skin. In that case, the number of dermal papillae that elevate up into the epidermis is reduced. Their presence ensures a proper nourishment of the epidermis through diffusion of nutrients. The wavelike course of the DEJ prevents skin delamination, which is why the aged skin is less resistant to mechanical injuries (34). Fibroblasts are responsible for the production of collagen, elastin, and extracellular matrix proteins, creating the spatial network of the dermis. In the intrinsically aged skin, fibroblasts become large and irregularly shaped, with signs of oxidative damage and decreased proliferation ability. In that case, the quantity of two main structures of the dermal tissue (collagen and elastin) is reduced. The aged skin loses strength and resiliency, so wrinkles and sagging are clinically manifested. Type I and III collagen show structural abnormalities and disorganization as a response to the increased activity of matrix metalloproteinases (MMPs). Physiologically, degradation of collagen is regulated by MMPs (mainly MMP-1, -3, -9) (35). Epidermal keratinocytes and dermal fibroblasts are the major source of MMPs in the skin. However, the activity of MMPs is regulated by tissue inhibitors of metalloproteinases (TIMPs). TIMP-1, TIMP-2, TIMP-3, and TIMP-4 are main protease inhibitors of MMPs (28). Their amount in the photo-aged and intrinsically aged skin is reduced, so the imbalance between MMPs and TIMP accelerates the progressive collagen fragmentation. The levels of MMP-1, -2, -3, -9, -10, -11, -13, -17, -26, and -27 are elevated in the aged human skin (11, 28, 35). Extrinsic and intrinsic sources may generate ROS that serve as a major stimulus increasing the MMP levels in the aged skin (35). ROS have various inhibitory or stimulatory roles in cell signaling. ROS spread senescence and activate signal pathways, such as a nuclear factor kappa-light-chain-enhancer of activated B cells (NF-κB) or mitogen-activated protein kinases (MAPK). The MAPK pathway is a major source of the transcription factor activator protein 1 (AP-1) that regulates the transcription of MMP1, MMP3, MMP9, and MMP12. AP-1 increases the expression of MMP1, MMP3, MMP9, and MMP12, leading to the collagen fragmentation in the skin. AP-1 also decreases the signaling of transforming growth factor-*β* (TGF-β), which is responsible for maintaining the structural integrity of the dermal connective tissues. NF-κB is another pathway activated by ROS. It is responsible for the upregulation of MMP-1 and MMP-3 in dermal fibroblasts (28, 35). Aging fibroblasts produce more ROS, generating oxidative stress, which further increases the expression of MMPs. Proteases, induced during photo-aging, lead to an impairment of the dermal fibroblast function, accumulation of cross-linked collagen, and a loss of dermal elasticity. The progressive ECM degradation is correlated with a reduction in the size of fibroblasts and their senescence stage (36). In the aged skin, the number of micro-vessels in the superficial dermis and within the dermal papillae decreases. They become small and lack architectural complexity. Due to those changes, blood flow and nutrient exchange are reduced. Body temperature decreases and the skin becomes pale (33). Moreover, with age the hypodermis loses subcutaneous fat, contributes to wrinkling and sagging of the skin (8). In particular, the mass of dWAT declines rapidly in older individuals (37). From middle age, the distribution of adipose tissue transitions from subcutaneous depots (SAT) to visceral depots (VAT). The loss of subcutaneous fat tissue leads to sunken cheeks and periorbital areas as well as sagging nasolabial fat (30, 38). The redistribution of WAT promotes the adipocytes hypertrophy. Large and mature adipocytes in VAT contribute to the development of metabolic diseases in elderly people (30). Compared to WAT, BAT mass remains unchanged until old age. The brown adipocytes become hypertrophied with enlarged lipid droplets (37). BAT-thermoregulatory properties declines with age, correlating with a decrease in the metabolic rate. The risk of obesity increases because, in contrast to the thinning of the hypodermis with age, other fat depots increase (39) (Figure 1).

Immunosenescence, as the deterioration of the immune system and its ability to respond effectively to harmful challenges, is associated with skin aging. The accumulation of senescent cells drives chronic inflammation and metabolic dysfunction, which deteriorates the aging process. Immunosenescence, together with chronic systemic inflammation, is called inflammageing. The skin contains several types of immune cells that work together to keep an active immuno-protective property of the skin. For instance, Langerhans cells, dendritic cells, macrophages, monocytes, natural killer (NK) cells and T cells coordinate immune responses in the skin (40). In the aging skin, there is an imbalance between inflammatory and immune reactions (41). Senescence cells are resistant to natural processes of elimination and apoptotic pathways by the immune system. Immune cells, after immunosenescence, undergo a series of changes. Every aging cell secretes proinflammatory factors, termed the senescence-associated secretory phenotype (SASP). The major components of the SASP factors in senescent fibroblasts are interleukins (IL), chemokines, and growth factors, that may exhibit a harmful impact on neighboring cells and the local tissue environment. In the case of chronological aging, in tissues an excessive secretion of the insulin-like growth factor (IGF-1), tumor necrosis factor-*α* (TNF-α), transforming growth factor *β* (TGF-β), insulin-like growth factor binding proteins (IGFBPs), IL-6, IL-1β, IL-2, IL-1, IL-18, MMP1, MMP3, MMP10, MMP12, and E2 prostaglandin have been noticed (11, 40–42). Fibroblasts from the chronologically aged skin increase the production of cysteine-rich protein 61 (CCN1), which is the cause of the unbalance in collagen homeostasis leading to the skin dysfunction (8). The receptors on NK cells, T cells, and monocytes/macrophages are able to recognize some of the SASP components, which affects other immune cells and causes more pro-inflammatory cytokines to be further released and age-related pathological symptoms to be exacerbated (42). Dermis fibroblasts support the healthy skin function, but the increasing number of senescent fibroblasts generates the inflammageing phenotype. That is why the levels of inflammatory cytokines are elevated in the aging skin (41).

Another obvious symptom associated with aging is hair loss or alopecia. Hair follicle shrinks and its regenerative potential decreases because of the wane activity of hair follicle stem cells. As a result, hair shafts become narrow and short, creating a “thinning” hair appearance. Some follicles are thought to completely lose the regeneration ability and stop hair production. Hair graying is still another sign related with age. In the young skin melanocytes transmit pigment into keratinocytes that differentiate to create the hair shaft. If the melanocyte stem cells have lost their activity, melanocytes cannot be formed. Pigment in hair follicle keratinocytes is absent and hair with gray or white shafts are only produced (43, 44).

### 3.2 Extrinsic factors driving skin aging

As internal aging affects the whole body, the external aging of the skin is limited to environmentally exposed skin parts, such as face, neck or the dorsal side of hands. In those exposed surfaces, symptoms like deep wrinkles, skin laxity, roughness, pigmentation disorders, such as lentigines, or visible blood vessels under the skin surface, known as telangiectasia, predominate (45). The major factor responsible for external aging is ultraviolet (UV) radiation, which accounts for more than 80% of facial aging (6). That is why extrinsic aging is also named photo-aging. There are three types of UV radiation, classified according to their wavelength, biological activity, and the ability to penetrate the skin. Short-wave UVC (200 ∼ 280 nm) with the strongest mutagenicity is fully blocked by the ozone layer and does not reach the earth’s surface. Medium-wave UVB (280 ∼ 320 nm) is biologically active with a strong mutagenic ability. Mostly UVB is filtered by the atmosphere and cannot penetrate beyond the superficial skin layers (6). UVB acts in the epidermal layer, causing keratinocytes and melanocytes to damage, but can penetrate to papillary dermis. UVB rays induce the production of MMP-1 by epidermal keratinocytes. It indirectly affects changes in the dermis, causing the breakdown of collagen fiber (45). Long UVA wavelengths (320 ∼ 400 nm) have a weak mutagenic potential but a strong penetration ability, which may damage the dermis and even subcutaneous tissues (6). Changes in those layers seem to be mainly responsible for the progression of photo-aging. While the UVA light is penetrating the skin, it is concurrently absorbed by cellular chromophores, such as a melanin, bilirubin, heme, or porphyrin. They collect photons, which leads to their excited state, called the singlet excited state. That initiates a series of reactions, during which products may interact both with DNA and molecular oxygen, leading to DNA modification or the production of ROS. The accumulation of damages in the dermal fibroblast mitochondrial DNA (mtDNA) generates oxidative stress and ROS production, leading to skin aging (46). Indeed, the UV-irradiated skin exhibits a thickened epidermis, mainly in the stratum corneum. The pores of the skin are large and more visible, filled with horny material. The skin surface exhibits roughness and yellow discoloration because of the excess amount of horny layers. In the case of the keratinocyte DNA damages, epidermal cells activate signaling pathways that lead to apoptosis. It results in the decreased keratinocyte activity and slower renewal ability. In the lower epidermal layers the number of atypic and apoptotic cells increases. Basal keratinocytes show irregularity. It affects the overall epidermis appearance. UV may also stimulate melanocytes to cause an intensified melanin synthesis and form age spots. Alterations in NMF have also been observed in response to the UV exposure. The reduced content of ceramides and fatty acids on the skin surface damages the lipid barrier of the epidermis, making skin rough (33).

**Figure 2:**
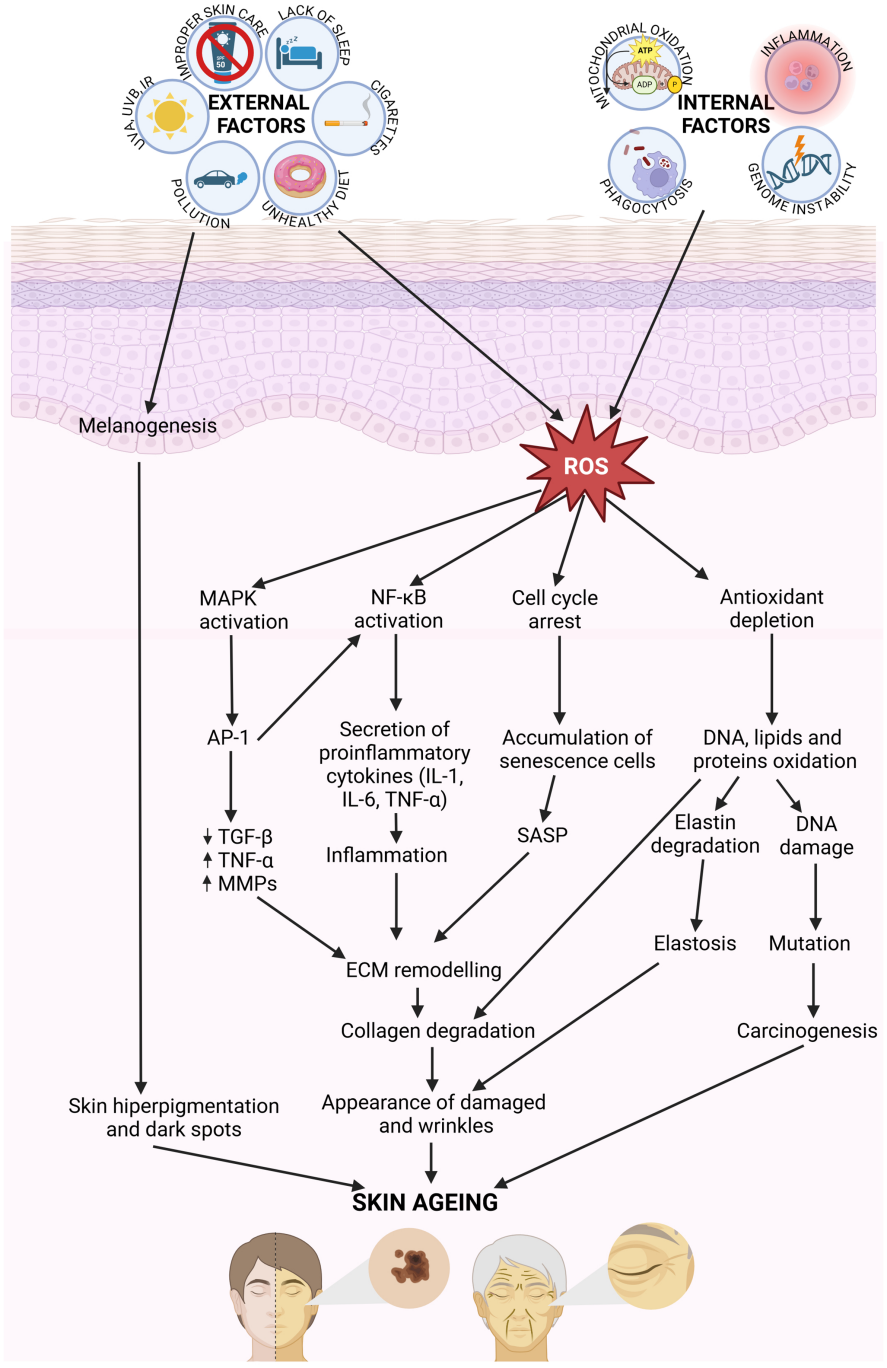
Molecular mechanisms involved in skin aging. Internal and external factors generate ROS, which activating the signaling pathway MAPK, NF-κB, and AP-1, involving in cytokines secretion. That affect for ECM remodeling. Collagen and elastic fiber break down, and skin become damaged and wrinkles. ROS results in the oxidation of DNA, lipids and proteins leading to damages in cellular function (created with BioRender.com).

Extrinsic skin aging is additionally related to degenerative changes in the dermis, including collagen deficiency or improper organization of collagen bundles. A dramatic decrease in the amount of type I collagen, along with a concurrent increase in the quantity of type III collagen, makes skin much weaker and less flexible. The number of fibroblasts in the dermis is slowly decreasing with age. Production of new collagen and elastin fiber slows down, the decomposition of whitch, as the consequence of the MMP activity, is accelerated (33). The main histopathological sign of photo-aging is elastosis—the accumulation of abnormal, thickened, and fragmented elastic fiber. The elastotic lesions in the upper dermis make skin thicken and wrinkle. There is a direct connection between the exposure to ROS, UV radiation, and structural damage to fibrillin microfibrils in elastic fiber. Fibrillin microfibrils are rich in UV chromophores that may be directly damaged by UV radiation, leading to the impairment of elastic fiber configuration (25, 28). The microcirculation is also affected by the sun exposure. Blood vessels become dilated and twisted. Firstly, their walls are thick, but as the destruction proceeds, they finally become thin. The vessel changes manifest themselves on the skin surface as telangiectasia (6, 8) (Figure 2).

Apart from the UV spectrum, natural sunlight also contains near-infrared radiation [(IRA) (770–1,400 nm)] and visible light, which contribute to skin aging as well (46). IRA deeply penetrates skin, reaching the dermis and subcutaneous tissue. It leads to collagen degradation and wrinkle formation. Visible light, especially blue light, affects the overall skin appearance. There is no evidence that blue light directly causes skin to wrinkle but there are studies that show the relationship between blue light and an induction of pigmentation lesions in the aging skin (45). Results of the clinical research clearly show that sunscreens are effective in diminishing the relapse of pigmentation disorders after the blue light exposure. Only indirectly may blue light affect the dermis lesions by inducing the MMP-1 expression (45, 47).

Extrinsic skin aging may also result from air pollution. Traffic-related particulate matter and soot, gasses such as nitrogen dioxide (NO_2_), and ground–level ozone are the factors that correspond with the unhealthy skin pigmentation and wrinkle formation. The traffic-related particulate matter is especially responsible for hyperpigmentation. On the other hand, ozone causes alterations in the stratum corneum, reduces the amount of natural antioxidants and generates stress, which leads finally to wrinkle formation (32).

Cigarette smoking has specific damaging effects on the skin. Cigarette smoking induces skin oxidative stress, which contributes to premature skin aging that manifests itself through skin wrinkling, particularly around the mouth, the upper lip and eyes (45). Tobacco smoke contains 3,800 constituents. Polycyclic aromatic hydrocarbons (PAHs), that contribute to the activation of the aryl hydrocarbon receptor (AhR) signaling pathway, are the most numerous ones. AhR activates the transcription of genes coding for proteins involved in growth control, ECM proteolysis regulation or cytokines/ROS production. PAH 2,3,7,8-tetrachlorodibenzo-p-dioxin (TCDD) and trans-retinoic acid elevate the MMP-1 expression in fibroblasts (48). MMP-1 induces the degradation of collagen and elastic fiber, leading to the imbalance between biosynthesis and degradation in the dermal connective tissue metabolism. Smoking decreases the synthesis of type I and III collagen in the skin and promotes the fragmentation of elastic fiber. Fibrillin microfibrils become more numerous and shorter (25, 49). That leads to an imbalance in the ECM homeostasis. The dermal structure breakdown and wrinkles appear (45). Smoking causes thickening of the skin, especially the stratum corneum, and leads to deterioration of skin pigmentation (50).

Other factors that cause premature aging include unhealthy diet and alcohol consumption. Excessive alcohol intake leads to skin dehydration. In consequence, skin loses proper elasticity, and sags, dries, and wrinkles. Blood in blood vessels beneath the skin surface is dilate, causing redness and flushing (50). Nutrition levels and eating habits also have a certain effect on skin aging. Food is a source of monosaccharides that, in high amounts, may lead to the production of advanced glycation end-products (AGEs) in the body. AGEs have been shown to promote protein cross-linking, oxidative stress, and inflammation (51). All those factors contribute to structural lesions in the dermal tissue, related to the collagen and elastin fiber breakdown, wrinkle formation and skin sagging. There is evidence of the association between a high content of sugar and fried or grilled products in a diet, with higher levels of AGEs in the skin (50). Avoiding food that increases the AGEs content, such as donuts, barbecued meat, and dark-colored soft drinks may be an effective strategy to prevent sugar-related skin lesions (51). Sugar metabolism is strongly linked with fat metabolism. A high-fat diet (HFD) may in a similar way cause morphological degradations in the dermis and damage the elements of ECM. Daily consumption of fatty products may promote skin inflammation by enhancing the expression of inflammatory factors, for example, cytokines such as interleukin-1β (IL-1β) and IL-18. A HFD also activates the NF-κB pathway that is another source of inflammatory reactions and pro-inflammatory cytokines (52).

In order to reduce harmful effects of AGEs supplied both from external and internal reactions, a daily diet should contain a high amount of antioxidants, for example resveratrol, *α*-lipoic acid, L-carnitine, vitamins, tea polyphenols, curcumin, flavonoids or silymarin (50, 51). Food-derived antioxidants reduce oxidative damage, inhibit collagen degradation and inflammatory reactions by means of regulating the activity of MMPs, cytokines, and NF-κB or MAPK proteins (50). Calorie restriction has a positive impact on both lifespan and health span. It is a dietary intervention where total caloric intake is reduced but sufficient nutrition is maintained (53). Dietary restriction prolongs lifespan by reducing the production of ROS, limiting oxidative damage, and increasing the DNA repair capacity. Calorie restriction has a positive effect on glucose metabolism, lipid profile, and blood pressure reducing the development of age-related diseases (54).

Sleep deprivation may increase ROS production leading to premature aging. It also may cause skin barrier impairment and intensify skin dryness (55). The network of nerves, and mechanical and chemical receptors in the human skin provides for a permanent interaction with the Central Nervous System (CNS). It makes skin particularly sensitive to the effects of emotional stimuli, including stress. The stress response activates two main pathways, the Sympathetic Nervous System (SNS) and the Hypothalamic–Pituitary–Adrenal (HPA) axis (56). As a result, catecholamines (norepinephrine and epinephrine) are released from the SNS, and corticotropin-releasing hormone (CRH)—from hypothalamus in the HPA axis. CRH induces the production of adrenocorticotropic hormone (ACTH) in the pituitary gland that subsequently triggers the adrenal glands to release cortisol. The release of neuropeptides and neurohormones impacts the skin by means of activating the endocrine, neurologic, and immune systems (56). A chronic exposure of cells and ECM components to catecholamines enhances oxidative stress and ROS production. Differentiation of epidermal keratinocytes declines, reducing the integrity of the skin barrier. A high level of cortisol inhibits collagen biosynthesis, leading to skin sagging and wrinkling. A chronic lack of sleep and a stressful lifestyle may lead to skin redness, dryness, dullness, and heightened sensitivity (55).

Improper skin care may also trigger premature skin aging. The lack of sunscreens in a daily skin care is the most common problem. Application of sunscreens to uncovered parts of the skin ensures protection against harmful UV rays. Such photo-protection may reduce the progression of skin aging. Application of antioxidants, such as coenzyme Q10 or vitamins A, C, E, ferulic acid, ensures the same preventive effect (57). The presence of antioxidants in cosmetics prevents oxidative stress and enhances DNA repair in skin cells, reducing inflammation and disrupting wrinkles formation, to the effect of slowing down the process of skin aging. It is now believed that only a combination of sunscreens and antioxidants provides a full UV protection (7). Young and healthy skin requires an effective skin barrier. Application of moisturizers prevents dehydration and penetration of irritants (57). Sunscreens, antioxidants, and moisturizers are the first step in home-based skin rejuvenation formulations. There are many natural and sustainable ways that successfully prevent excessive and premature dermal aging. Small changes in life habits are enough to enjoy young and healthy skin for many years (8, 45).

Dietary restriction prolongs lifespan by reducing the production of ROS, limiting oxidative damage, and increasing the DNA repair capacity. Calorie restriction has a positive effect on glucose metabolism, lipid profile, and blood pressure reducing the development of age-related diseases (58). A group of drugs called senotherapeutics indicate a potential to remove senescent cells. Senotherapeutics are categorized into two groups: senolytic and senomorphic drugs. Senolytic drugs have the ability to induce the death of senescent cells and selectively eliminate the SA-β-gal activity. Senolytic drugs, such as Bcl-2 family inhibitors [Navitoclax (ABT-263) and (ABT-737)] reduce the SASP secretion (59). Senomorphic drugs (e.g., rapamycin) have the ability to inhibit the SASP phenotype of aging cells. Rapamycin inhibits the mTOR pathway involved in the synthesis of SASP and reduces oxidative stress in the photoaged skin. Senomorphic drugs do not directly intact the senescent cell but minimize the negative effects of cellular senescence on surrounding tissues. Rapamycin is a major senomorphic agent with the anti-aging potential (59).

## 4 Theories of skin aging

### 4.1 Somatic DNA damage theory

The somatic DNA damage theory suggests that aging is a consequence of accumulation an unrepaired DNA mutation. That genomic instability may accumulate in healthy cells throughout life, decreasing the cellular function (60). The DNA defects may include chemical modifications, nucleotide insertions or deletions, nucleotide substitutions, cross-linking with other nucleotides and single- or double-stranded DNA breaks. Such events affecting the genome architecture may lead to genome instability and may also trigger the DNA damage response (DDR). The DDR inhibits the cell cycle progression and activates signaling pathways such as damage repair, apoptosis or cellular senescence (61, 62). Biological organisms generate a wide variety of DNA repair mechanisms that promote proper DNA replication during cell division and removal of DNA damage. When there is a lot of accumulated DNA damage, the proper response is impossible. That leads to apoptosis or cellular senescence. Every aging cell has a disturbed metabolism and secretes pro-inflammatory factors, known as SASP. SASP is responsible for the generation and spread of cellular senescence (62). Fibroblast’s SASP consists of increased concentrations of interleukins, chemokines, and MMPs. Human fibroblasts express TNF-*α*, related to inflammation and cells apoptosis. A high concentration of those factors contributes to the premature skin aging, emergence of age-related diseases and malignancies, including squamous cell carcinoma (63).

### 4.2 Theory of limited cell division

In the early 1960s, Leonard Hayflick and his colleagues discovered replicative aging in *in vitro* cell cultures. They observed that cells were able to divide a limited number of times before they underwent apoptosis. It means that each cell has a programmed number of mitoses in its life cycle, called Hayflick Limit. The closer they are to the limit, the more signs of aging they show (36, 64). Hayflick discovered that skin fibroblasts could divide 40–60 times before their senescence, while the lifespan of typical human keratinocytes is around 15–20 population doublings (36). That theory of a limited number of cell divisions is strongly linked to the telomere theory (64, 65).

### 4.3 Telomeres theory

Telomeres are guanine rich DNA sequences that play a vital role in genome protection. Those structures at the termini of eukaryotic chromosomes protect the genome from unnecessary recombination, nucleolytic degradation or interchromosomal fusion (64, 66). The main problem is that telomeres naturally shrink with each cell division. As replication cycles proceed, even 55 base pairs per year are lost from the end of chromosomes (66). That leads to critically short telomeres and, eventually, a dramatic reduction in chromosome length. That “end replication problem” may be mitigated by the presence of telomerase. The DNA at the 3′ end of the chromosome has a repetitive sequence known as the telomeric repeat. Telomerase, as an enzyme, extends the 3′ end by means of synthesizing multiple copies of the telomere repeat. That ensures telomere stabilization and mitigates telomeres shortening during cell proliferation. Telomerase has been recognized to be a key determinant of human health and longevity. Its insufficiencies are associated with premature aging and stem cell renewal disorders. With age, the telomerase activity declines but the natural process of telomere shortening proceeds. That leads to the loss of vital information, resulting in cell death (67). The latest studies suggest that not only critically short telomeres but also long and defective telomeres contribute to cellular senescence and age-related diseases. The accumulation of irreparable damage in telomeres triggers a response leading to cell death. In the aged skin, keratinocytes, melanocytes, and fibroblasts are the examples of the length-independent telomere dysfunction. Telomeres of normal length trigger the telomere DDR response, contributing to the induction of replication stress (68). The Hayflick’s limit theory and the telomeric theory are connected with disturbed homeostasis and collectively contribute to cellular senescence (69).

### 4.4 Membrane theory

According to the membrane theory, as the body ages, the composition of cell membranes changes, making them stiffer. Lipids constitute an essential element of the cell membrane. They contribute to the flexibility and fluidity of the membrane. With age their amount decreases much more than water content, making the membrane transport difficult. Toxic substances accumulate in cells. One of them is lipofuscin (LF). It is lipopigment formed by lipids, metals, and misfolded proteins. LF cannot be degraded or cleared by exocytosis because of its polymeric and highly cross-linked nature. That is why it accumulates within the lysosomes and cell cytoplasm in senescent cells, especially in nerve cells, cardiac muscle cells and skin, being an intracellular marker of aging (70, 71).

### 4.5 Mitochondrial theory and free radical theory

Mitochondria are the cell center of oxidative metabolism and the main site of ROS production. Their activity is crucial in the mitochondrial theory of aging. In the human body approximately 90% of oxygen is consumed by mitochondria (72). Redox signaling modulates cellular physiology and functions. ROS play a dual role in a cell. On the one hand, they are crucial to maintain biological processes and body health. On the other hand, an increased production of ROS causes functional damage to fundamental cellular components throughout the body, including proteins, lipids, and nucleic acids. The natural antioxidant defense declines with aging, which, combined with the increased production of ROS, leads to a state of an oxidative stress. A chronic oxidative stress plays an important role in cells senescence (73). It is proved that dysfunction of mitochondria and impairment of their morphology have been correlated with aging. Besides the increased ROS production, mitochondria of senescent cells show genomic instability, unbalanced NAD+/NADH ratio, and a Ca^2+^ overload and mitochondrial DNA mutations (74). As it turns out, mitochondria constitute the main target of oxidative damage caused by an excess amount of ROS. Phospholipids and proteins in the mitochondrial membrane are especially sensitive to the ROS-induced lipid peroxidation and protein oxidation. A high ROS production in older species also contributes to DNA damage (72). ROS disturb the cell respiratory chain and reduce ATP production, leading to a decrease in tissue oxygenation (75). The mitochondrial theory of aging is strongly linked with the free radicals’ theory. ROS are highly active molecules. Hydroxyl radical (^•^OH), superoxide anion (O_2_^•-^), hydrogen peroxide (H_2_O_2_), singlet oxygen, alkoxyl and peroxyl radicals are the main ROS representatives (73). Cellular metabolic processes provide for the natural presence of ROS. However, ROS may also be produced as a response to various environmental stimuli such as ultraviolet light, ionizing radiation, and air pollutants. Long–term exposure to exogenous factors leads to an excess ROS production and may contribute to cellular oxidative damage. Intensity of that state depends on a type of ROS and a related production rate, efficiency of the endogenous defense mechanisms, and dietary intake of antioxidants, and food supplements (76). The endogenous antioxidant system is composed of enzymatic and non-enzymatic components, and its prime function is to prevent the formation and interaction of ROS with cell components. The enzymatic antioxidants include superoxide dismutase (SOD), catalase (CAT), glutathione peroxidases (GPx1–GPx8), glutathione transferase (GST) and glutathione reductase (GR). The majority of non-enzymatic components are low-molecular-weight substances, including uric acid, lipoic acid, melatonin, metal chelators (transferrin and lactoferrin), ubiquinone (coenzyme Q10), transition metal ions (zinc, copper, and selenium), vitamin E, vitamin C, *β*-carotene, and polyphenolic compounds (77, 78). A high level of free radicals in the absence of effective antioxidant mechanisms may disrupt the cellular redox balance, known as oxidative stress. ROS damage vital cellular macromolecules, including membrane lipids, proteins, and nucleic acids (79) (Table 2). Lipid peroxidation is the reaction between free radicals and membrane lipids, especially polyunsaturated fatty acids (PUFAs). Lipoperoxidation end-products (aldehydes, ketones, and hydroxyperoxides) cause disintegration of cell membranes and increase their permeability. The most mutagenic and toxic products of lipid peroxidation are malondialdehyde (MDA) and 4-hydroxy-2-nonenal (4-HNE) (80). In the epidermis, lipid components of sebum and ceramides as well as PUFAs in the cell membrane constitute the main target of peroxidation. ROS activate NF-κB, a complex cellular response in keratinocytes. NF-κB regulate the expression of telomerase genes, cellular proliferation, inflammation, and anti-apoptotic factors associated with cellular longevity (81). Proteins are highly abundant in cells, extracellular tissues, and body fluids, which makes them the most susceptible components to oxidation. The ROS activity damages the structure of proteins by oxidizing numerous sites on the side chains and backbones of proteins. Protein carbonylation is an irreversible post-translational modification leading to protein unfolding and aggregation (82). Carbonyl (CO) groups may be formed by direct oxidation of lysine, arginine, threonine, and proline residues. In result, carbonyl derivatives are formed: 2-pyrrolidone from proline, glutamic semialdehyde from arginine and proline, *α*-aminoadipic semialdehyde from lysine, and 2-amino-3-ketobutyric acid from threonine (82). Oxidative protein modifications disrupt skin proteins, both structural and enzymatic, which is responsible for the aging process (77, 83).

**Table 2:** Oxidative damage to proteins, lipids and nucleic acids.

	Lipid peroxidation	Protein oxidation	Nucleic acids oxidation
Mechanism	ROS reaction with PUFAs from membrane lipids and other lipid components from sebum and ceramides leads to chain breakage and increase in skin membrane permeability	ROS reaction with lysine, arginine, threonine, and proline residues leads to fragmentation of the peptide chain and site-specific amino acid modification	ROS reaction with pyrimidine and purine bases leads to DNA-protein crosslinking and modification of bases
Products of reaction	MDA, 4-HNE, F2-isoprostanes	2-pyrrolidone from proline, glutamic semialdehyde from arginine and proline, α-aminoadipic semialdehyde from lysine, 2-amino-3-ketobutyric acid from threonine; protein carbonyls, AGEs, AOPPs, oxLDL	8-oxoG, DNA strand breaks
Disturbances in cellular signaling	NF-κB pathway activation	MAPK pathway activation	Mutagenesis and carcinogenesis

ROS, reactive oxygen species; PUFAs, polyunsaturated fatty acids; MDA, malondialdehyde; 4-HNE, 4-hydroxy-2-nonenal; DNA, deoxyribonucleic acid; AGEs, advanced glycation end-products; AOPP, advanced oxidation protein products; OxLDL, oxidized low-density lipoprotein; 8-oxoG, 8-oxo-7,8-dihydroguanine.

It has been shown that oxidative stress activates the MAPK pathway in skin keratinocytes. MAPK kinases interact with various substrate proteins regulating cellular activities (proliferation, differentiation, inflammatory responses, and apoptosis) (81). ROS compromise the function of tyrosine-related protein 1 (TRP1) involved in melanogenesis, which contributes to oxidative stress-induced degradation of melanocytes. Free radicals also enhance the expression of proteinases responsible for ECM remodeling in skin, especially serine proteases and MMPs. They are responsible for the degradation of skin collagen and wrinkles formation (81). The nucleic acids constitute still another biological structure susceptible to the adverse impact of ROS. The hydroxyl radical causes DNA damage through oxidation of pyrimidine and purine bases. Such modification of nucleotide bases leads to 8-oxo-7,8-dihydroguanine (8-oxoG) production, the one-electron oxidation product of guanine (84). That modification of the genetic material may lead to mutagenesis, and carcinogenesis (79). A permanent oxidative damage to the DNA bases of genes coding for ECM components, such as collagen and elastin, leads to their reduced expression in the aged skin. DNA damage, protein carbonylation, lipid peroxidation, and damage to mitochondrial DNA increase with advancing age. Those types of oxidative damage have been proposed as biomarkers of aging (79).

### 4.6 Cross-linking theory

**Figure 3:**
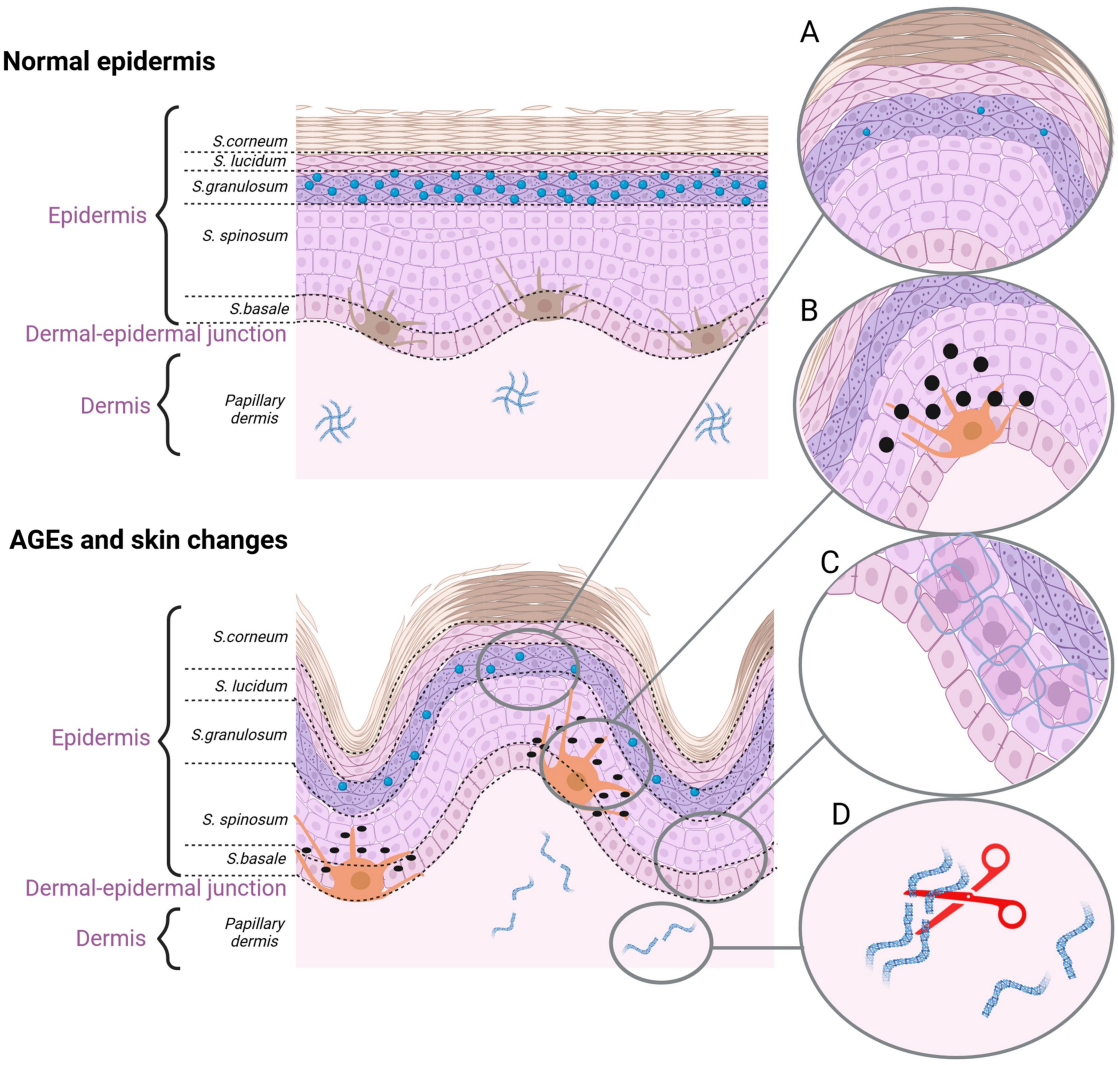
The effect of AGEs on the skin aging. **(A)** Dehydrated epidermis. AGEs reduce the content of ceramide and cholesterol. **(B)** Pigmentation lesions. AGEs promote the production of melanine. **(C)** AGEs damage the keratinocytes. **(D)** AGEs cause fiber deformation and disruption of the ECM organization (created with BioRender.com).

The last theory of aging is the cross-linking theory, also known as the glycation theory. Oxidative stress and excess amount of ROS play a key role in catalyzing the formation of AGEs. AGEs are heterogeneous particles derived from the non-enzymatic reactions between sugars and proteins. The formation of AGEs disturbs the protein structure due to covalent cross-linking, leading to alterations in cellular functions, cell damage or even death (85, 86). AGEs may be divided into three categories according to their cross-linking structure and fluorescence properties. The first class consists of fluorescent and cross-linked AGEs with such examples as glyoxal-lysine dimmer (GOLD), methyl-glyoxal-lysine dimmer (MOLD), 3-deoxyglucosone-derived lysine dimer (DOLD) or pentosidine (PEN). The second class includes non-fluorescent and cross-linked AGEs, for example, imidazolium dilysine. In the third group there are non-fluorescent and non-cross-linked types of AGEs, such as N-carboxymethyl-lysine (CML), N-carboxyethyl-lysine (CEL) and pyrraline (87). Another classification divides AGEs in respect of their molecular weight. CML or PEN are representatives of low molecular weight AGEs (LMW-AGEs), the mass of which does not exceed 12 kDa (86). In the group of high molecular weight AGEs (HMW-AGEs), GOLD, MOLD and DOLD, the mass of which is greater than 12 kDa, prevail (86). Glycation, as a random and non-enzymatic process, has to be distinguished from glycosylation that is an enzymatic, fully controlled reaction. Formation of AGEs is a complicated long-term molecular process, known as the Maillard reaction (MR). Protein molecules with the slow turnover rate, such as tissue collagens, constitute the most common target for the formation of AGEs. In the first stage of the MR, nonstable products, known as Schiff bases, are generated. The electrophilic carbonyl group of a reducing sugar reacts with free amino groups, especially lysine or arginine residues. Rearrangement of unstable bases leads to formation of stable ketoamines known as Amadori products. Those reversible and unstable chemical products may react with peptides or proteins in an irreversible way in order to form protein cross-links. Moreover, those compounds may participate in oxidation, dehydration or polymerization reactions, leading to the formation of many AGEs (78, 85). Currently, at least 20 AGEs have been detected in the human skin, as derived both from exogenous and endogenous factors (86). Exogenous stimuli, such as a high-sugar, high-fat or high-protein diet, cigarette smoke, UV, and air pollution, contribute to AGEs accumulation in skin. Endogenous AGEs come from physiological age-related metabolism or from diseases associated with chronic metabolic disorders such as diabetes and chronic kidney disease (88). It has been shown that a long-term oxidative stress promotes formation of endogenous AGEs (89). Several studies have demonstrated that circulating glycotoxins and dietary AGEs lead to alterations in cell signaling and functioning (86). It is not surprising that AGEs affect all levels of the skin (Figure 3). AGEs accumulation in the epidermis results in the keratinocytes rearrangement and loss of the epidermis thickness. The epidermis becomes thin and dehydrated due to epidermal atrophy triggered by CML (90). AGEs also reduce the content of skin ceramide and cholesterol, in effect of which the skin barrier is destroyed. Moreover, under those conditions, melanocytes increase the melanin production, making skin cells prone to pigmentation lesions. Products of the sugar and protein reaction in keratinocytes aggregate in the skin and manifest as the skin yellowness (86). AGEs also disturb the dermis’s ECM because of the increase in the activity of MMP-1, MMP-2, and MMP-9, leading to the fiber deformation. In addition, AGEs cross-linking with collagen and elastin change biomechanical properties and function of fiber. Formation of the collagen triple helix structure may also be difficult due to an improper aggregation of monomers. Thus, collagen fiber becomes stiff and less flexible (78, 85, 86). Collagen deformation reaches almost 80% of all the tissue deformation caused by the products of AGEs (86).

Glucosepane, CML, pentosidine and CEL are the most important and numerous AGEs in the skin. Concentration of glucosepane is the highest from all the AGEs, which makes glucosepane a key factor in increasing stiffness and hardness of collagen fiber (91). The presence of CML in the skin is regarded as a major indicator of collagen glycation (78, 85, 86). AGEs influence the tissues through their interaction with specific receptors. RAGE (receptor for advanced glycation end products) is one of the most classical cell surface receptor for AGEs. It is expressed in various skin cells including fibroblasts and keratinocytes (92). AGEs, by binding with RAGEs, may promote oxidative stress by increasing the level of ROS. Capability of the antioxidant defense system decreases with age, accounting for a reduced amount of SOD, catalase, GSH and ascorbic acid (85). The binding AGE/RAGE activates various signaling pathways that induce, for example, the activation of the NF-κB pathway or MAPK leading to the production of pro-inflammatory cytokines and ROS (78, 85). RAGE signaling is also involved in the MMP-9 activation in keratinocytes, growth factor secretion or fibrosis (overgrowth and hardening of various tissues). RAGE activation in melanocytes promotes melanogenesis (92). However, in response to a harmful action of AGEs, organisms activate glycation defense systems. There are many diverse signaling pathways that protect tissues from glycation-induced damage. The glyoxalase system, fructose amine 3-kinase (FN3K) repair enzyme, the ubiquitin-proteasome system (UPS) and the autophagy system constitute the main metabolic pathways against AGE-mediated cytotoxicity. They act at various stages of the ROS formation. For example, the glyoxalase system and FN3K prevent the formation of AGEs precursors. Moreover, enzyme FN3K provides for the reverse of chemically unstable Amadori products, prevents proteins cross-linking. Finally, the task of UPS and the autophagy system is to eliminate AGEs from organism. The activity of the defense systems against AGEs decreases with age, giving rise to the accumulation of AGEs-induced damage in skin (78, 85, 86).

## 5 Biomarkers of cellular senescence and skin aging

Cellular senescence is the primary aging process at the cellular level, associated with the loss of proliferative capacity. Every cell indicates a functional and morphological change that may be used as markers of senescence. Due to the multiplicity of cellular mechanisms that may drive a replicative arrest, there is a variety of potential biomarkers that may be considered to be biological age predictors. In clinical practice, studying the biomarkers provides the knowledge how advanced the physiological changes in the aging skin are. It is conducive to understand the phenomenon and mechanism of such a complex process in the human body. A number of multilayered and multifaceted aging markers have been described. According to the type of changes, the biomarkers may be divided into phenotypic and molecular ones (93).

Physical function and anthropometry are the most practical measurements among non-molecular phenotypic biomarkers of aging. A walking speed and standing balance, chair stand, grip strength (a predictor of frailty) and muscle mass, fat indices, body mass index, or waist circumference are well known physical functional measurements. With age, a decline in the physical function is frequently observed. Muscle loss, progressive frailty and physiological changes in the bodily organs are often associated with age-dependent chronic disorders (94). Quantitative external human facial features are correlated with phenotypic biomarkers of aging. Measurements of mouth and nose width, or eye corner droop are based on three-dimensional (3D) facial images, and may quantify the biological age (93).

The molecular group of biomarkers consists of routine laboratory biomarkers and high-throughput analyses. The routine biochemistry and immunology findings are widely measured in accredited clinical laboratories according to standardized methods. There are many routine age-related biomarkers in the blood. TNFα, C-reactive protein (CRP) and inteleukins constitute the examples of inflammation-linked biomarkers. Elevated levels of those compounds in the blood of older individuals contribute to the development of age-related diseases such as type 1 diabetes mellitus and coronary artery disease (94). Another group of biomarkers refers to lipid levels. Lipids in the blood may affect aging in the same way as they may undergo aging themselves. Levels of total cholesterol, HDL-cholesterol (high-density lipoprotein), LDL-cholesterol (low-density lipoprotein) and triglycerides vary with age, contribute to the cardiovascular disease progression. Age-dependent alterations may occur in the kidneys and liver. Creatinine, cystatin C, urea, bilirubin and albumin are markers of such damaging alterations in those bodily organs. Other routine laboratory biomarkers of aging may be, for example, glycated hemoglobin (Hba1c), and glucose (fastened or tolerance), insulin and C-peptide (the cleaved part of proinsulin), or iron level (94).

The largest study was conducted on 852 healthy individuals aged 30–98 years old, who were categorized into four groups based on age: young group, middle-age group, old group, and very old group. Given the blood plasma and urinary analyses, researchers screened 108 variables associated with physical characteristics, homeostatic state, and cardio-vascular function. Seven most significant composite indicators used for predicting the rate of aging were selected: (I) markers related with the atherosclerotic progression and hemodynamic state of carotid artery: intima-media thickness of the carotid artery (IMT), end-diastolic velocity (EDV); (II) markers related with the arterial compliance: arterial pulse pressure (PP); (III) markers related with the kidney function: cystatin C (CYSC); (IV) markers related with the cardiac diastolic function and the state (or rate) of left ventricular relaxation: ratio of peak velocity of early filling to atrial filling (E/A), mitral valve annulus lateral wall of peak velocity of early filling (MVEL); (V) markers related with the inflammatory indication: fibrinogen (FIB). The results show substantial differences in the biomarkers content in each group—with age, there was an increase in IMT, PP, FIB, and CYSC content, while EDV, E/A, and MVEL decreased. The analysis of those factors could be applied to predict the rate of aging (95, 96).

**Figure 4:**
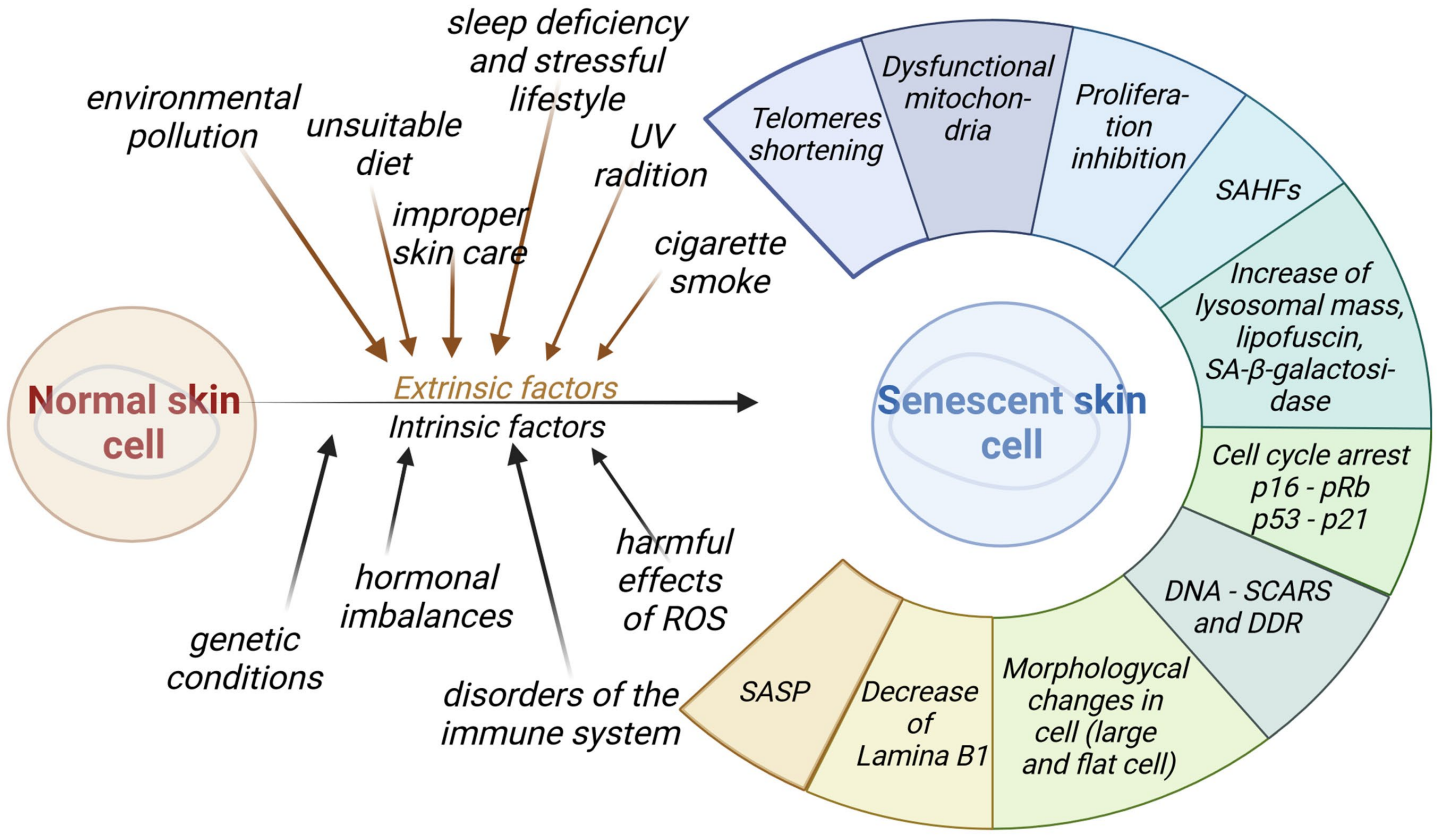
Impact of intrinsic and extrinsic factors for skin senescence. Aged cells generate a wide range of diverse functional and morphological changes that may be used as biomarkers of senescence (created with BioRender.com).

The second type of molecular biomarkers are high-throughput analyses. They provide opportunities for better understanding of the pathological process of aging at the DNA, RNA or cellular level. Biomarkers of that type are classified into special groups. The first class accounts for changes at the level of the cell structure and morphology: enlarged and flattened cells with some impairment in the nuclear membrane, a high activity of senescence-associated *β*-galactosidase (SA-β-gal), accumulation of lipofuscin, and an increased lysosomal mass, and cells granularity. The second group of biomarkers pertains to the changes related to the cell cycle regulation, including inhibition of proliferation, cell cycle arrest, and increased levels of cell cycle inhibitors (p16INK4a, p21CIP1, p53). DNA and heterochromatin tags, such as the presence of DNA-SCARS, SAHF, PML-NB, shortening of telomeres, downregulation of lamina B1, constitute another group of biomarkers. Within the last group of biomarkers the senescence-associated secretory phenotype (SASP) is induced—releasing a large number of growth factors, cytokines, chemokines, and proteases (10, 11, 97, 98). Figure 4 summarizes the functional and morphological changes in skin senescence following exposure to internal and external factors.

### 5.1 Morphological changes in aging cells

The first group of senescence markers includes markers associated with structural changes in aging cells. An increase in size and flattening of the shape may be observed in senescent cells. Those morphological changes may be easily detectable using light microscopy or flow cytometry (98). The size of senescent fibroblasts has been found to increase up to nine times (99). The increasing size has also been confirmed in aged keratinocytes and in human melanocytes after UVB—induced senescence. The nuclei of senescent skin cells have shown hypertrophy. It has been clearly demonstrated by the study, where human dermal fibroblasts were examined at early (<10% life span completed) or late (>95% life span completed) passage. In both cultures the nuclear area was measured digitally and was 255m^2^ at an early stage and 293m^2^ at a late passage (99). A new, hopeful biomarker of senescence, associated with morphological changes, may derive from alterations in the plasma membrane protein expression (99). The plasma membrane consists of an impermeable phospholipid bilayer and proteins immersed in it. Plasma membrane proteins participate in the communication between the inside and outside of the cell, regulating the movement of ions, water, or nutrients across the plasma membrane. Membrane proteins participate in almost all physiological processes. Their differential modification, aberrant expression, or any abnormal activity are linked to many common diseases. That makes them the efficient potential as biomarkers (100). Surface proteins may be readily identified and isolated from a phospholipid bilayer, maintaining the membrane integrity. That would be useful to rapidly detect the senescent cells, to define the interactions of those cells with the microenvironment and to explain the clearance mechanisms of senescent cells. Aging cells show alterations in the plasma membrane protein expression. Dipeptidyl-peptidase 4 (DPP4) is a highly expressed surface protein in senescent human diploid fibroblasts. The presence of DPP4 triggers the NF-κB signaling pathway, leading to inflammatory reactions and elimination of senescent cells (101). Secretory carrier membrane protein 4 (SCAMP4) is a secretory protein involved in membrane transport, promoting SASP secretion in human fibroblasts. SCAMP4 is positively correlated with the presence of IL-6 and IL-8 in senescent fibroblasts. Dermal cells exhibit an altered secretome and undergo morphological, and metabolic alterations, playing a primary role in driving skin inflammation (101). Intercellular adhesion molecule-1 (ICAM-1), a cluster of differentiation 36 (CD36), or malondialdehyde-modified vimentin also constitute surface glycoproteins highly expressed by a variety of senescent cells, including human fibroblasts (98, 101).

### 5.2 Increase in lysosomal mass, accumulation of lipofuscin and high activity of senescence-associated β-galactosidase

Aging skin cells are often accompanied by an increase in the lysosomal activity, which may be detected by enzymatic staining. There is an increase in the lysosomal mass associated with the accumulation of old lysosomes and increased activity of lysosomal biogenesis. Identification of lipofuscin content may be used as a main biomarker of skin lysosome accumulation. Lipofuscin has autofluorescence properties that may be used for detecting its content in cells, tissues and body fluids. Fluorescence-based methods or selective staining with Sudan black B facilitate the identification process (98). The LF fluorescence emission demonstrates a very wide spectra—from 400 to 700 nm, with a maximum around of 578 nm. Thus, for the purpose of proper LF detection and quantification, fluorescence microscopy needs to be used (71). As an insoluble material, lipofuscin consists of proteins, lipids, and sugars, that are highly oxidized and cross-linked (99). LF cannot be degraded or cleared through exocytosis and it accumulates within the lysosomes and cell cytoplasm in replicative senescence. Accumulation of lipofuscin has been detected both in senescent fibroblasts and in the basal layers of the aged epidermis (71, 99). Morphological lysosomal changes are mostly associated with an increased activity of the lysosomal enzyme, senescence-associated-galactosidase (SA-β-gal). SA-β-gal is a biomarker closely related to the senescence and cellular proliferation status. The enzyme activity has been confirmed in human keratinocytes and fibroblasts, that undergo replicative senescence. The same results have been obtained in *in vitro* conditions, in skin samples, and in the cells isolated from elderly people. However, SA-β-gal has some limitations and it cannot be accepted as specific enough to identify senescent cells by itself. It may detect senescence lesions only at pH 6.0, otherwise the results will be falsely positive even in non-senescent cells (6). In quiescent cells that do not divide but still retain the ability to re-enter cell proliferation, the activity of SA-β-gal also increases. Some stressful circumstances may lead to the intensification of that enzyme activity in cells. In response to such stressors as the UV light, ionization radiation, cigarette smoke or oxidants, the premature senescence in cultured fibroblasts, keratinocytes, and melanocytes cells has been confirmed. It is worth mentioning that not all senescent cells express SA-β-gal (98, 99). SA-*α*-fucosidase is another biomarker of senescence closely related with SA-β-gal. That enzyme activity increases with lysosomal activation during aging and it is more specific upregulation than in the case of SA-β-gal (98).

### 5.3 Increase in cells granularity

The increase in granularity of senescent fibroblasts results from the above mentioned intensification in the lysosomal activity. Accumulation of lipofuscin in the form of intracellular deposits especially affects the cell granularity level. In senescent cells the increase in granular content may be detected by means of the transmission electron microscopy and flow cytometry using the side scatter parameter (99).

### 5.4 Accumulation of dysfunctional mitochondria

During senescence, morphological and functional changes in skin mitochondria may be observed. Their high number and an elongated, enlarged shape are associated with a reduction in mitochondrial potential. Dysfunctional mitochondria constitute a major source of an increased ROS production in senescent cells. An excess number of ROS leads to a damage to the mitochondrial membrane, including lipid peroxidation. Genetic mutations in the mitochondrial DNA accumulate over time, leading to decreases in protein synthesis and energy production. Natural autophagy of mitochondria is disrupted, and the damage within the cell leads to the increased mitochondrial dysfunction. Studies of senescent human foreskin fibroblasts clearly demonstrate highly elongated and enlarged mitochondria that increase in number up to 90–94 times (72, 73, 99).

During stress, inflammatory or immune responses, mitochondria or their fragments may be released from cells as circulating mitochondrial DNA (ccf-mtDNA). Increased blood ccf-mtDNA levels have been associated with aging and age-related diseases such as type 2 diabetes or heart disease. Ccf-mtDNA content proportionally increases with age, which has been clearly revealed within the framework of the previous study. The positive correlation has been indicated for the increased amount of ccf-mtDNA in mature adults after the fifth decade of life (102, 103).

Nicotinamide adenine dinucleotide (NAD^+^) is crucial to energy metabolism, including glycolysis, tricarboxylic acid (TCA) cycle, and fatty acid oxidation. NAD^+^ is the major hydride acceptor in redox reactions, forming its reduced version, NADH. NAD^+^ may also undergo phosphorylation to form nicotinamide adenine dinucleotide phosphate (NADP^+^), which is also a hydride acceptor (104). Due to this, NADPH is produced, that is used for the purpose of protection against oxidative stress. The recent studies have linked the role of NAD^+^ with aging. A study conducted on a group of people aged 20–87 years has shown that the plasma levels of NAD^+^ decrease significantly with age. Aging-related diseases, including metabolic diseases, neurodegenerative diseases, or cancer are also associated with decreased levels of NAD^+^. NAD^+^ directly controls secretion of SASP, which affects cellular aging. The presence of dysfunctional mitochondria and an imbalanced NAD^+^/NADH ratio have been noted in the senescence, particularly in the case of replicative aging (105).

### 5.5 Inhibition of cell proliferation

Once the senescence program is initiated, skin cells become unable to proliferate and their number stay unchanged. Proliferating skin cells have a unique ability to incorporate bromodeoxyuridine (BrdU) or 5-ethynyl-2-deoxyuridine (EdU) into DNA, which may be detected by immunocytochemical methods—the ELISA technique or flow cytometry. Senescent cells have no such an ability to merge BrdU or EdU to DNA. They demonstrate low or absent DNA synthesis, which is a starting point to cell cycle arrest [84]. Another commonly used marker of proliferation is Ki67 protein that is exclusively expressed in proliferating cells. The presence of Ki67 may be detected through immunocytochemistry. In senescent cells there should be no expression of Ki67 (98).

### 5.6 Cell cycle inhibitors

As the cell cycle proceeds, an unexpected DNA damage may occur. In normal skin cells, a defense mechanism triggers a transient cell cycle arrest to repair the damaged DNA, and as the mission is complete, cell cycle is resumed. An excessive damage, irreparable lesions or a delayed DNA repair lead to cellular senescence or apoptosis (11). The cell cycle is regulated by cyclins, cyclin-dependent kinases (CDK) and cyclin-dependent kinases inhibitors protein (CDKI). Cyclins, as a group of related proteins, bind with enzymes CDKs to generate the cyclin-CDK complex that plays an important role in the control of cell division. An active complex will phosphorylate its protein, conduct the cell cycle progression, and activate target genes. DNA damage detection leads to the activation of the inhibitors of CDK. There are two CDKI protein families: the inhibitor of CDK4/6 (INK4) family, which includes p16INK4A, and kinase inhibitor proteins (CIP/KIP) comprising p21CIP1 (98, 99).

p21CIP1 (p21) is an essential protein for the initial phase of senescence during which cells stop dividing. p21 is expressed by a variety of senescence-inducing signals, for example, the direct transactivation of p53. p53 encourages cyclin-dependent kinase inhibitor 1 (CDKN1A) to encode cell cycle inhibitor p21Cip1. p21, as the main p53 effector, inhibits CDKs at either G1/S or G2/M checkpoints, repressing the cell cycle progression (21, 98). Interaction between p53—p21CIP1 is one of the major senescence–associated pathways that regulate the proliferative arrest. After detection of DNA damage, p53 activates p21CIP1, the expression of which increases dramatically in the initial phase but decreases in the further senescence progression. p21 inhibits the activity of CDK responsible for activation of retinoblastoma tumor suppressor protein, pRb. The lack of pRb inactivates pRb/E2F pathway to preclude DNA synthesis. pRb may be inactivated by both p21CIP1 and p16INK4A (17).

p16INK4A (p16) is a unique and specific marker of senescence, that is encoded by gene CDKN2A (cyclin-dependent kinase inhibitor 2A). As an inhibitor of CDK4 and CDK6, D-type cyclin-dependent kinases, it is involved in maintaining the phosphorylation of pRb. pRb, in the hypophosphorylated state, negatively regulates the E2F-transcription factor activities and represses the cell cycle progression—blocks cells in the G1 phase. p16INK4a is responsible for an irreversible stage of cells senescence, called senescence maintenance (98, 99). p16INK4A–pRb pathway leads to cell cycle arrest and cell senescence (17). p16INK4a affects pRb/E2F pathway in the same way as p21CIP1does, regulating the activation of Rb family proteins. In the retinoblastoma protein family, the main members associated with senescence are Rb/p105, p107, and Rbl2/p130. Especially Rbl2/p130 plays an important role in cell cycle arrest and senescence maintenance. It interacts with E2F-4, creating a complex that regulates the E2F target genes expression and represses the cell cycle progression—blocks cells in the G1 phase (6, 98, 99). Over-expression of p16 in young cells is related with senescence phenotypes. In mice, the removal of p16 delayed aging. p16 is present in various aging cells. Due to its easy detectability by means of Western blotting, it is considered a universal biomarker of cellular aging (6).

Proteins belonging to the p16-pRB and p53-p21 senescence induction pathways are universal biomarkers of cellular senescence. They may be easily determined by means of Western blotting (WB), Immunohistochemistry (IHC), and Immunofluorescence detection, and may be over-expressed in various senescent cells (6, 98).

### 5.7 DNA damage response and DNA-SCARs formation

Unrepaired DNA damage accumulates in cells through life. DNA alternations associated with skin aging include chemical modifications, nucleotide insertions or deletions, nucleotide substitutions, cross-linking with other nucleotides and single- or double-stranded DNA breaks (62). In order to sustain genome integrity, skin cells have evolved specialized DNA damage response (DDR) mechanisms. Senescent cells are characterized by a chronic DDR presence with an absence of DNA repair mechanisms. DDR may be identified through the permanent presence of such protein clusters as *γ*-H2AX (phosphorylated form of histone H2XA), 53BP1 foci, and activated ataxia-telangiectasia mutated (ATM) kinase. Double-strand DNA breaks, as important activators of DDR, attach the ATM to the site of DNA damage (106). That triggers the phosphorylation of histone H2AX and many other substrates, including the two principal checkpoint kinases—checkpoint kinases 1 (CHK1) and checkpoint kinases 2 (CHK2), which spreads the phosphorylation cascade and thus DDR signaling. It has been shown that permanent activation of DDR is the result of DNA damage in telomere sections. The occurrence of several lesions in telomere sections is a sufficient stimulus to induce aging and it could be used as a senescence marker (99). Damaged telomeres and a persistent unrepaired DNA damage lead to the accumulation of DNA-SCARS (DNA segments with chromatin alterations reinforcing senescence) in a cell nucleus. Those clusters contain proteins such as ATM, γH2AX and 53BP1, that constitute a hallmark of senescent cells. They modulate a local chromatin structure and support DNA repair. The occurrence of DNA-SCARS may be detected by means of immunocytochemical staining. ATM also phosphorylates a number of effector proteins that are not involved in DNA repair, for example, p53, CHK1, CHK2, and IKKγ. Their presence is also required to establish and maintain the secretory phenotype of senescent cells. All DNA-SCARS are dynamically formed distinct structures involved in the regulation of a complex senescent phenotype (62).

### 5.8 Shortening of telomeres

Telomeres shrinking is still another marker related to skin replicative aging. It may be measured using FISH (fluorescence *in situ* hybridization) and southern blotting techniques (98). It has been proven that basal cells in UV-exposed skin regions exhibit shorter telomeres in comparison to sun-protected regions. UVA irradiation induced the formation of 8-oxodG in DNA fragments containing the telomeric sequence GGG approximately 5 times more than in DNA fragments with non-telomeric sequences (107). With age, the telomerase activity, as a telomere protector, declines. That disrupts the telomere stability, leading to the telomere shortening. The human epidermis in sun-protected areas demonstrates little or no telomere loss with aging. It may result from the fact that keratinocytes in the basal layer express active telomerase (8, 40).

The telomere length and telomerase activity in lymphocytes of the immune system decline with age. Senescent T cells with the shortest telomeres and the lowest telomerase activity generate much more pro-inflammatory cytokines, which inhibits a proper immune response and spreads the senescence for neighboring cells (42).

### 5.9 Presence of SAHF

Senescent nuclei contain senescence-associated heterochromatin foci (SAHF). Those punctate heterochromatic domains are thick structures created as a result of chromatin condensation. SAHF may lock skin cells in a senescent state by repressing proliferation-promoting genes and the cell cycle progression. SAHF contain various markers, such as trimethylated histone H3 Lys 9 (H3K9me3) and heterochromatin proteins 1 (HP1α, *β*, and *γ*). They may be detected by means of staining with 40, 6-diamidino-2-phenylindole (DAPI) and could serve as senescence skin markers (98, 99, 108).

### 5.10 Downregulation of lamina B1

The composition of the skin nuclear membrane is changing during senescence and that may affect nuclear morphology and gene expression. Lamina B1 is a structural protein of the nuclear lamina. It has been shown that the level of lamina B1 is downregulated in skin cells undergoing replicative senescence and in the chronological aging. Such destabilization of nuclear integrity may affect the formation of cytoplasmic chromatin fragments associated with DNA damage. The decrease in lamina B1 levels has been found to be associated with the activity of two major senescence—associated pathways: p16-pRb and p53-p21. The content of lamina B1 in the nuclear membrane, as a valuable senescence marker, may be determined by means of the quantitative polymerase chain reaction (qPCR), immunohistochemistry methods (IF) or Western Blot WB (98, 99).

### 5.11 Induction of senescence-associated secretory phenotype

Senescent skin cells secrete immunomodulators, inflammatory cytokines, growth factors, chemokines and proteases commonly named as senescence-associated secretory phenotype (SASP) or senescence-messaging secretome (SMS). The secretion of SASP/SMS factors into the microenvironment of tissues is caused by DNA damage-induced senescence and may be detected by means of WB and ELISA (98). Senescent cells exhibit an altered secretome and undergo transcriptional, epigenetic, morphological, and metabolic alterations, playing a primary role in driving skin inflammation. Key elements of the SASP include the proinflammatory cytokines such as interleukin-6 (IL-6), interleukin-8 (IL-8), and interleukin-1α (IL-1α). In the chemokines group, both CC chemokine ligands (CCL family) and C-X-C motif chemokine ligand (CXCL family) members are present: CCL-2 (MCP-1), CCL-20 (MIP-3), CCL-7 (MCP-3), CXCL-4 (PF-4), CXCL1 (Gro-), and CXCL-12 (SDF-1) and GRO-α, CXCL-2, CXCL-3, and CXCL-5. SASP is also comprised of hundreds of protein and non-protein signaling molecules. Proteases, hemostatic factors, bradykinins, ceramides and ECM components are amount them. MMPs, such as MMP-1 and -3, constitute another important element of SASP. As the regulatory elements of senescence, they may cleave IL-8, IL-1 and other CXCL/CCL family chemokines (97, 109). SASP is an ambiguous marker of cellular senescence. It is nonspecific and may be secreted by non-senescent cells under some conditions. That is why SASP should not be used as a single biomarker. However, in *in vitro* research on senescent dermal fibroblasts and melanocytes, MMPs, chemokines receptors (such as CXCR2), and cytokines (such as IL-6 and IL-8) have been used as a marker. In *in vivo* conditions, the melanocytes level of IL-6 is elevated. MMPs are numerous in the chronologically aged and photo-aged skin (98, 109). For example, MMPs are produced by epidermal keratinocytes in response to the exposure of human skin to solar UV radiation. The events that activate SASP are linked with DDR. For example, the persistent DDR activity is required for the IL-6 and IL-8 induction (99).

### 5.12 Correlation between senescence of human dermal microvascular endothelial cells and skin biomechanical properties

Vascular endothelial cells make up a mere barrier between blood and tissues. The vascular endothelium lines the entire circulatory system, from big arteries to the smallest capillaries. The main function of HDMEC is to maintain homeostasis through the plasma and cells. They have the ability to generate new blood vessels but they are also important for inflammatory reactions, wound healing, and tissue growth. Vascular endothelial cells, similarly to the other cells, have a limited proliferation capacity. They undergo irreversible growth arrest or senescence and their functions are reduced with age. Aging of endothelial cells contributes to a decrease in the integrity and elasticity of blood vessels. The risk of inflammatory reactions increases, which may induce various age-related vascular diseases, such as atherosclerosis, hypertension, congestive heart failure, diabetes. Harmed blood vessels increase the risk of cancer metastasis (110).

In the studies of HDMEC senescence, aging-related biomarkers have been identified through various *in vivo* and *in vitro* experiments. Researchers from the Yonsei University College of Medicine have found such biomarkers as psoriasis-associated fatty acid-binding protein (FABP5), moesin, and Rho-GDP dissociation inhibitor (RhoGDI), thioredoxin domain-containing protein 5 (TXNDC5), Ras suppressor protein-1 (RSU1) and vimentin. A higher expression of those six skin aging biomarkers has been confirmed in aging human dermal fibroblasts (HDFs), human epidermal keratinocytes (HEKs), and HDMECs after SA-β-gal staining. Immunohistochemistry (IHC) and qRT-PCR methods have revealed that old skin have decreased protein levels of moesin, TXNDC5, RhoGDI and RSU1 and increased protein levels of vimentin and FABP5. The same researchers have verified the correlation of those six biomarkers with skin aging values, elasticity, skin density, and elastic recovery. They measured biomechanical skin properties using non-invasive methods. The elasticity of the facial skin was tested by means of a cutometer. The negative pressure created in the device deforms the skin, mechanically drawing the skin into the aperture of the probe, and after a defined time, releases it again. The resistance of the skin to the negative pressure (firmness) and its ability to return into its original position (elasticity) diminishes with age. In order to determinate skin thickness and density (both epidermal and dermal), an ultrasound imaging (Ultrascan) has been used. The ultrasound waves penetrate to a depth of 6-8 mm and are reflected differently by skin tissues (111). Then waves are transformed into electrical impulses, creating an ultrasound image. The last measurement of biomechanical skin properties has been based on the torsional method—Dermal Torque Meter (DTM). The probe consists of a rotating central disk separated by a 1 mm distance from a fixed outer guard ring. During application of the permanent load, the DTM measures the angular rotation of the disk and registers the rotation as angular deformation. After load discontinuation, the DTM detects the rotational recovery of the skin. All three methods show exactly that biomechanical properties, such as skin elasticity, skin density, and elastic recovery decrease with age. It has allowed to draw conclusions that protein levels of TXNDC5, RhoGDI, RSU1 and vimentin are all meaningfully correlated with the decreasing properties of skin values in all the three non-invasive tests. The moesin protein level has not been connected with the cutometer results, while the FABP5 protein level has not been correlated with the data obtained from the Ultrascan and DTM. The main result of the correlation between the non-invasive measurement devices and the molecular biological analysis results has been the selection of four biomarkers correlated with skin biomechanical properties. It has been TXNDC5, RhoGDI, RSU1, and vimentin. They can successfully be used as biomarkers of skin aging (111).

TXNDC5 and vimentin are strongly expressed in fibroblasts. A high level of those proteins is associated with NF-κB signaling pathway and is a source of aberrant inflammatory responses and pro-inflammatory cytokines, such as IL-6, IL-8, IL-13, and TNF-*α*. Inflammation caused by the TXNDC5 or vimentin activity has been identified as a main trigger for organs fibrosis, including skin. Fibrosis is a consequence of an abnormal tissue repair mechanism. Activated fibroblasts deposit excessive ECM components, leading to disruption of the tissue architecture and organ dysfunction. Skin fibrosis disturbs biomechanical properties of the skin (112, 113).

### 5.13 Skin fluorescence as marker of long-term AGEs accumulation in aging skin

Accumulation of AGEs occurs both in the intrinsic and extrinsic skin aging. Manifestation of those products in the connective tissues disturbs ECM adhesion proteins, such as collagen and elastin, resulting in the loss of skin elasticity and stiffness of collagen fiber. It is known that AGEs cumulate also in blood vessels, muscles, bones, and joints, contributing to induction of age-related diseases, such as cardiovascular disease, sarcopenia, fracture, and osteoarthritis. The assessment of skin autofluorescence (SAF) is a non-invasive technique to estimate AGEs accumulation in the skin. The measurement is performed by means of AGE reader. The device utilizes the fluorescent properties of AGEs and estimates their accumulation in skin, given the emission and reflection spectrum. The results are converted through a software program into numerical values (AU). A higher SAF score in AU indicates a higher level of tissue AGEs (78, 85, 114).

The research, conducted on participants from the Rotterdam Study, shows the connection between the tissue level of AGEs and their role in the development of frailty in the elderly adults. Researchers have compiled the results of SAF in skin with its association for physical frailty phenotype. They have defined frailty as a set of symptoms of diminished strength, endurance, and a reduced physiological function. Special measurements were used for evaluating weakness, weight loss, slowness and exhaustion of each of 2,521 participants at the medium age of 74 years (67–81). About 56% of them had at least 2 components of frailty. The results suggest that increasing the frailty status is related with higher SAF values, older age, female gender and as well as type 2 diabetes mellitus. In the study population all the individuals with either a low physical activity, connected with weakness and exhaustion, have had higher SAF values. That means that skin autofluorescence correlated with AGEs accumulation is positively associated with a frailty index. Thanks to the possibility of measurement of skin SAF, this factor may be useful as a biomarker of skin aging (114).

### 5.14 Clinical significance of aging biomarkers

Cellular senescence is a complex process resulting in a progressive loss of cell functionality and regenerative potential. The identification of cellular, molecular, and physiological markers may contribute to a better understanding of the complexity of the aging process (115). According to the American Federation for Ageing Research, biomarkers of cellular senescence must predict the rate of aging and life-span better than the chronological age (99, 115). They need to control the basic process of cellular senescence, not the effects of such changes. Potential biomarkers of aging have to be active both in humans and in laboratory animals, in order for them to be tested firstly at animals. They must have a repeatable potential for many laboratory tests, without any harmful effects for the person concerned (99, 115).

Recent studies have provided supporting evidence for the potential value of some biomarkers in predicting and monitoring age-related diseases, such as Alzheimer’s disease, diabetes, osteoarthritis, and cancer (116–119). Age is also a major risk factor for developing life-threatening disease, including vascular disease (120, 121). Reduced elastic fiber, myocardial hypertrophy or fibrosis, chronic inflammation and lipofuscin accumulation constitute indicators of cardiac aging. Biomarkers of inflammation, such as CRP, IL-6 and fibrinogen, are also positively correlated with vascular diseases related with aging (121).

Potential biomarkers of skin aging are presented in Table 3. However, there is no specific and non-invasive indicator for skin aging, that could reliably assess the rate of senescence in the epidermis or dermis. There is also a lack of blood markers or other easily accessible specimen to measure skin senescence. That still requires more attention (94). However, few studies have reported that some biomarkers related with skin aging may be detected in the body fluids. The age-related skin dysfunction leads to an increase in the levels of cytokines and growth factors in the bloodstream, including TNFα, IL-1α, IL-19, and IL-23 (122). The levels of many markers associated with aging are altered in the skin wash; a reduction in indicators of collagen synthesis, specifically epidermal growth factor (EGF) and fibroblast growth factor (FGF), has been reported (121). The impaired anti-inflammatory response (IL-1Ra) and innate immunity (interferon alpha-2, IFN-α2) has also been noted (121). A promising value in the aging biomarker group is provided by TXNDC5, RhoGDI, RSU1, and vimentin. A higher expression of those markers has been confirmed in aging HDFs, HEKs, and HDMECs. Researchers have strongly linked the levels of TXNDC5, RhoGDI, RSU1, and vimentin with impaired biomechanical skin properties such as skin elasticity, skin density, and elasticity restoration in the aging skin (111). AGEs accumulation in intrinsic and extrinsic skin aging is another clear indicator linked with the degradation of the aging ECM. There is a strong correlation between the presence of AGEs in the skin and the diminished strength, endurance, and the reduced physiological function of the human body with age (85, 114). Assessment of AGEs fluorescence in the skin may serve as a non-invasive indicator of the body’s aging. Further studies are needed on larger patient populations to assess the diagnostic potential of those biomarkers, in particular their sensitivity and specificity.

**Table 3:** Potential biomarkers of skin aging.

Biomarker	Diagnostic material	Analytical technic	References
Enlarged, flattened cell morphology	Cultured cells	Light microscopy, FC	Hartmann et al. (94), Kudlova et al. (98)
Increase in lysosomal mass	Cultured cells	Enzymatic staining	Kudlova et al. (98)
Accumulation of lipofuscin	Cultured cells	SBB, GL13	Hartmann et al. (94), Kudlova et al. (98)
SA-β-gal	Cultured cells	Enzymatic staining	Kudlova et al. (98)
Increase in cell granularity	Cultured cells	Electron microscopy, FC	Csekes and Račková (99)
Dysfunctional mitochondria (increased blood ccf-mtDNA levels) and an imbalanced NAD+/NADH ratio	Living cells, mitochondrial DNA	Various methods	Hartmann et al. (94)
BrdU or EdU absence in DNA	DNA	Staining incorporation, IF	Kudlova et al. (98)
p16INK4A	Blood	WB, IHC, IF	(94), (98), et, al., Hartmann, Kudlova
DNA-SCARs formation	DNA	IF	Hartmann et al. (94)
Chromatin modification	DNA	Chromatin remodeling assays	Hartmann et al. (94)
8-oxoG in DNA telomeric fragments	Saliva	qPCR, FISH	Hartmann et al. (94)
SAHF (H3K9me3, HP1*α*, *β*, and *γ*)	Cultured cells	DAPI/Hoechst, confocal microscopy, IF, IHC	Hartmann et al. (94), Kudlova et al. (98)
Lamin B1	Cultured cells	IHC, WB, IF, qPCR	Hartmann et al. (94), Kudlova et al. (98)
SASP: interleukins IL-6, IL-8, IL-1α, and chemokines CCL-2 (MCP-1), CCL-20 (MIP-3), CCL-7 (MCP-3), CXCL-4 (PF-4), CXCL1 (Gro-), CXCL-12 (SDF-1) CXCL-2, CXCL-3, and CXCL-5	Serum, plasma	ELISA, EDTA, WB	Hartmann et al. (94)
Presence of HDMEC senescence biomarkers: FABP5, moesin, RhoGDI, TXNDC5, RSU1 and vimentin	Tissue	qPCR, IHC	Kim et al. (111)
Accumulation of AGEs	Tissue	SAF	Waqas et al. (114)

ccf-mtDNA, circulating mitochondrial DNA; NAD, nicotinamide adenine dinucleotide; NADH, reduced version of nicotinamide adenine dinucleotide; BrdU, bromodeoxyuridine; EdU, 5-ethynyl-2-deoxyuridine; SA-β-gal, senescence-associated β-galactosidase; DNA-SCARs, segments with chromatin alterations reinforcing senescence; 8-oxoG, 8-oxo-7,8-dihydroguanine; SAHF, senescence-associated heterochromatin foci; SASP, the senescence-associated secretory phenotype; HDMEC, Human Dermal Microvascular Endothelial Cells; FABP5, fatty acid-binding protein; RhoGDI, Rho-GDP dissociation inhibitor; TXNDC5, thioredoxin domain-containing protein 5; RSU1, Ras suppressor protein-1; AGEs, advanced glycation end-products; FC, flow cytometry; SBB, sudan black B staining; GL13, sensitive lipofuscin staining; WB, Western Blot; IF, immunofluorescence; IHC, immunohistochemistry; DAPI, flow cytometry combined with 4’,6-diamidino-2-phenylindole; qPCR, quantitative polymerase chain reaction; SAF, skin autofluorescence.

It is necessary to differentiate between a potential biomarker and a reliable biomarker. The most useful biomarkers in clinical practice require validation, a formal analytical assessment of sensitivity, precision, accuracy, or detection limit. The analytical validation accounts for a biomarker’s technical performance and ensures consistent measurements (123). Clinical validation demonstrates the usefulness of a biomarker and reliability of such an indicator of the patient’s clinical status (95). A valuable biomarker must have a repeatable potential for many laboratory tests, without any harmful effects for the individuals (99, 115). A biomarker with a repeatable potential in independent experiments becomes a much more authentic diagnostic instrument. The data arising from promising biomarker studies have been shown not to be reproducible. Experimental conditions, marker heterogeneity, or measurement errors may lead to reduced reproducibility (95). Finally, a major drawback of biomarker research is the high cost of the process. The choice of the biomarker for research purposes should be based not only on scientific considerations but also on available financial resources. The complexity of the results, the number of patients in the study or the reproducibility determine all the costs (124).

Skin aging is a direct manifestation of the body’s aging. Monitoring of aging biomarkers could ensure the qualitative and quantitative analysis of senescent cells, including skin cells, which allows us to better understand the physiological and biochemical changes of the aging skin (6). Our review indicates that a single biomarker is inadequate to provide a clear and unambiguous evidence of skin aging. Certain conditions may interfere with the diagnostic value of biomarkers, giving false results, such as comorbidities, diet, physical activity, cosmetics and dietary supplements used. Therefore, further research is needed, ranging from the elucidation of the causes and mechanisms of skin aging to the validation of biomarkers in clinical practice. New non-invasive biomarkers of aging should also be sought to replace studies on cellular models.

## 6 Conclusion

In this review, we have summarized the current knowledge on the aging process, especially skin aging and biological age predictors. The most recognizable implications of tissue aging manifest themselves on the skin. Accumulation of endogenous (gene mutation, cellular metabolism or hormonal agents) and exogenous factors (UV, environmental pollutants, unsuitable diet) trigger the aging process that begins at the cellular level. Senescence of dermal fibroblasts and keratinocytes is the main cause of skin aging that occurs as a decline in the integrity and function of the skin. Skin laxity, roughness, pigmentation disorders and age spots, wrinkles, telangiectasia, hair graying and loss are physiological aging symptoms. Aging is also associated with the development of a wide variety of diseases affecting all the bodily organs and their systems over an extended period of time. That proves how complex and multifaceted process it is. Phenotypic (a non-molecular) and molecular types of biomarkers have been described. The phenotypic data include the physical function of body—the walking speed, standing balance, chair stand, grip strength and muscle mass, body mass index, or waist circumference. Molecular types of biomarkers ensure the specific understanding of the aging process by means of examining a number of its markers in routine laboratory tests or through high-throughput analyses. Molecular biomarkers of aging are those associated with the accumulation of metabolic products specific to cells throughout aging, such as the accumulation of lipofuscin, SA-β-gal, and cell cycle inhibitors. Other markers involve the presence of DDR, DNA-SCARs and SASP. Biomarkers may be detected by means of the immunocytochemical methods, such as WB, ELISA or flow cytometry. Aging is heterogeneous and requires many diverse biomarkers to be used for evaluating the age stage. In order to avoid cross-interactions between biomarkers, they have to be obtained from different organs in the body, to highlight the systemic changes that occur with aging. In the recent years, research has been conducted to determine the relationship between molecular biomarkers of aging and biomechanical parameters (non-molecular biomarkers) of aging skin. The study has verified that TXNDC5, RhoGDI, RSU1, and vimentin are the promising biomarkers, correlated with decreasing elasticity and density of the skin with age. In the last years, scientists have also paid more attention to AGEs accumulation in cells and tissues. They have linked AGEs presence in skin with the diminished strength, endurance and the reduced physiological function of the human body with age. Biomarkers are significant diagnostic indicators of the aging process but their efficacy needs to be verified in more diverse skin aging studies.
